# The Interplay between Mitochondrial Morphology and Myomitokines in Aging Sarcopenia

**DOI:** 10.3390/ijms22010091

**Published:** 2020-12-23

**Authors:** Vanina Romanello

**Affiliations:** 1 Veneto Institute of Molecular Medicine, Via Orus 2, 35129 Padova, Italy; vanina.romanello@unipd.it; Tel.: +39-04-9792-3268; Fax: +39-04-9792-3250; 2 Department of Biomedical Sciences, University of Padova, Via G. Colombo 3, 35100 Padova, Italy

**Keywords:** mitochondrial dynamics, fusion, fission, mitophagy, sarcopenia, FGF21, GDF15, mitokines, myokines

## Abstract

Sarcopenia is a chronic disease characterized by the progressive loss of skeletal muscle mass, force, and function during aging. It is an emerging public problem associated with poor quality of life, disability, frailty, and high mortality. A decline in mitochondria quality control pathways constitutes a major mechanism driving aging sarcopenia, causing abnormal organelle accumulation over a lifetime. The resulting mitochondrial dysfunction in sarcopenic muscles feedbacks systemically by releasing the myomitokines fibroblast growth factor 21 (FGF21) and growth and differentiation factor 15 (GDF15), influencing the whole-body homeostasis and dictating healthy or unhealthy aging. This review describes the principal pathways controlling mitochondrial quality, many of which are potential therapeutic targets against muscle aging, and the connection between mitochondrial dysfunction and the myomitokines FGF21 and GDF15 in the pathogenesis of aging sarcopenia.

## 1. Introduction to Sarcopenia: Implications for Skeletal Muscle Aging

In addition to its locomotion role, skeletal muscle has a critical role in whole-body metabolism and myokine-mediated interorgan crosstalk, controlling health and disease progression in distant tissues [1,2]. Its dysfunction is a major determinant of poor prognosis and reduced quality of life. Sarcopenia is an age-related skeletal muscle disease recognized by the World Health Organization with an International Classification of Diseases 10 code [3]. It is defined as a progressive and generalized decline in muscle mass and force. Lean mass loss begins at a slow rate around the fifth decade (almost 1% of reduction each year) to accelerate by 70 years of age, resulting in 30–50% of lean mass reduction [4]. Muscle strength deficiency precedes muscle loss, declining three times faster than muscle mass [5], suggesting that age-related muscle dysfunction depends not only on muscle size but also muscle quality [6]. Sarcopenia, the major cause of functional deterioration in older people, has several adverse clinical outcomes and socio-economic consequences. Postural and locomotion muscle groups’ functional insufficiency leads to loss of mobility and independence with impairment in daily living activities [7] and is the primary cause of falls and fall-related injuries in older humans [8]. The dysfunction of key respiratory muscles in the elderly increases the incidence of respiratory failure [9]. Moreover, general muscle deficiency in sarcopenic individuals can disrupt the intertissue communication and the systemic metabolic control exerted by muscles, increasing the risk of developing age-related diseases [2] such as obesity, diabetes, cardiovascular disease, cancer, and all-cause mortality [2,8,10,11]. Thus, sarcopenic patients are more prone to immobility and/or hospitalization, which exacerbates muscle dysfunction [12] and accelerates the sarcopenic progression due to the impossibility of completely recover muscle mass and strength in aged humans [13] and animals [14,15]. Sarcopenia is, on average, present in 5–13% of individuals over 60 years old and 50% of persons over the age of 80 years [4]. It is a significant emerging problem because people worldwide are living longer. According to the National Institute on Aging (NIH), by 2050, the world’s population aged 65 and older is expected to total 2 billion, up from 900 million in 2015. Due to poor knowledge of the mechanisms involved, there are no drugs to counteract sarcopenia. Sarcopenia is a complex multifactorial process. Nine primary hallmarks that contribute to the aging process were specified including, genomic instability, telomere attrition, epigenetic alterations, loss of proteostasis, deregulated nutrient sensing, cellular senescence, stem cell exhaustion, altered intercellular communication, and mitochondrial dysfunction [16]. Mitochondrial number, function, and morphology are tightly associated with muscle mass and function [17], and a functional mitochondrial network is critical for preserving skeletal muscle throughout the lifespan. Accordingly, mitochondrial dysfunction has a central role in aging sarcopenia [16]. Here, we discuss (1) the relevance of a functional interconnected mitochondrial network in skeletal muscle homeostasis; (2) the age-related alterations in mitochondria quality control pathways in skeletal muscle; and (3) the role of the myomitokines fibroblast growth factor 21 (FGF21) and growth and differentiation factor 15 (GDF15) in health and disease, and the link of muscle mitochondrial morphology and FGF21 and GDF15 with healthy or unhealthy aging. 

## 2. A Highly Interconnected Mitochondrial Network Is Tailored to Promote Energy Distribution and Support the Myofiber Function

Skeletal muscle is a plastic tissue that can undergo adaptive changes in its size, fiber type composition, or functional properties in response to several stimuli, such as nutritional status or contractile activity. According to the functional demands, skeletal muscles recruit the most suitable myofibers to modulate the expected response. Mitochondrial oxidative phosphorylation (OXPHOS) is the primary source of the high energy required during intense skeletal muscle contraction [18]. Thus, long-lasting contractions require the recruitment of oxidative myofibers characterized by high mitochondrial content, increased reliance on OXPHOS, and fatigue resistance. On the other hand, since glycolytic fibers have low mitochondrial content, decreased reliance on OXPHOS, and are fatigable, they can generate a high-intensity activity for short periods. Mitochondrial health is crucial for muscle plasticity and the fulfillment of skeletal muscle roles such as force production, metabolism, thermoregulation, signaling, and myokine production. The content and the distribution of mitochondria are finely tuned to achieve the myofiber’s specific function. Mitochondria are organized in a physically and functionally highly interconnected mitochondrial network in skeletal muscle [19,20,21]. High-resolution microscopy studies identified four different mitochondrial morphologies within the myofiber mitochondrial network: the paravascular mitochondria (PVM), I-band mitochondria (IBM), fiber parallel mitochondria (FPM), and cross-fiber connection mitochondria (CFCM). All these mitochondria are highly interconnected, allowing a rapid energy distribution in the form of electrical conduction from the periphery to the central part of the myofiber. PVMs are primarily involved in the generation of the proton-motive force near the capillaries. Since PVMs are coupled to IBMs, the proton-motive force is delivered to IBMs via the mitochondrial reticulum to produce ATP near the ATPase activity in the contractile apparatus. Thus, the physically and electrically connection of the mitochondrial network enables the muscle to respond almost instantaneously to energy requirements changes [20]. Mitochondrial shape and function are strictly connected. The mitochondrial network can morphologically adapt to the cellular energy requirements by continuously alternating fusion and fission events, ultimately maintaining a healthy mitochondrial population. Fission fragments the network and segregates dysfunctional or damaged components of the mitochondrial network. On the contrary, fusion leads to elongated mitochondria with increased interconnectivity into the network and facilitates the redistribution of metabolites, proteins, and mitochondrial DNA (mtDNA). Moreover, the fusion between healthy and damaged organelles allows diluting the damaged material into the healthy network, avoids the accumulation of dysfunctional mitochondria, and maintains their overall function [22]. The mitochondrial connectivity within myofibers is fiber-type dependent since oxidative fibers have higher fusion rates than glycolytic fibers [23]. Moreover, mitochondrial fusion in skeletal muscle is necessary to adapt to the cell’s specific functional needs and support skeletal muscle myofibers contractile function [24]. Accordingly, the mitochondrial network configuration in oxidative fibers has a grid-like pattern, with elongated mitochondria parallel and perpendicularly oriented to the muscle contraction axis. In contrast, the mitochondrial network in glycolytic fibers is fragmented and perpendicularly oriented to the muscle contraction axis [23,25]. Thus, the plastic nature of skeletal muscle converges on the capacity of the mitochondrial network morphology, arrangement, and connectivity to adapt to each fiber type’s specific functional needs.

## 3. Mitochondrial Quality Control Pathways Are Critical to Maintain Mitochondria and Muscle Health

**Figure 1:**
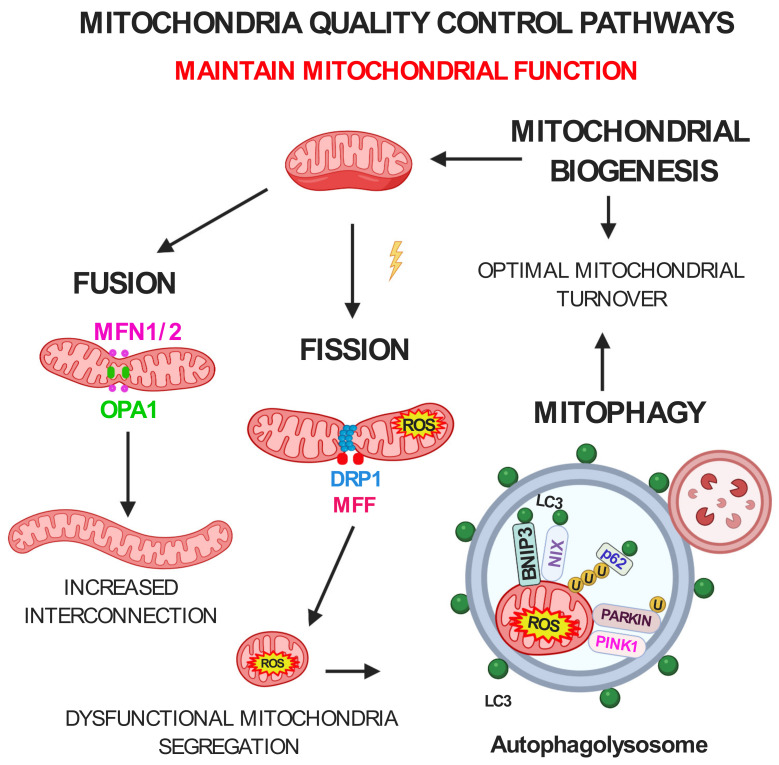
Mitochondria quality control pathways maintain mitochondrial function. Mitofusin 1 and 2 (MFN1/2) and optic atrophy protein 1 (OPA1) mediate mitochondrial fusion to produce an interconnected healthy mitochondrial network. Mitochondrial fission depends on dynamin-related protein 1 (DRP1) and mitochondrial fission factor (MFF) proteins. Fragmented dysfunctional mitochondria are removed by mitophagy. Bcl2/adenovirus E1B 19 kDa protein-interacting protein 3 (BNIP3) and NIP3-like-proteinX (NIX) are mitophagy receptors that bind to microtubule-associated protein 1 light chain 3 (LC3) to tether mitochondria to the autophagosome. PTEN-induced kinase 1 (PINK1) accumulates on depolarized mitochondria surface, where it phosphorylates ubiquitinated outer mitochondrial membrane (OMM) proteins and Parkin. Parkin will further promote the ubiquitination of the OMM proteins. The p62/SQSTM1 adaptor can recognize the ubiquitinated proteins to initiate mitophagy. U: ubiquitin. Figure was created with BioRender.com

As already discussed, a functional mitochondrial network in skeletal muscle is critical to support the metabolic demands imposed by contraction, energy expenditure, and general metabolism. Defective mitochondria cannot be cleared by cellular division due to the postmitotic nature of skeletal muscle. Thus, in muscle the preservation of the mitochondrial network integrity relies on the coordination of mitochondria quality control systems, which are activated, according to the degree of mitochondrial damage, ranging from a local repair to a dysfunctional organelle’s whole degradation [26]. Among the defensive strategies, continuous rounds of mitochondrial biogenesis and fusion are balanced by opposing processes of mitochondrial fission and mitophagy, the selective degradation of mitochondria via autophagy, to ensure the fusion of newly formed mitochondria into the mitochondrial network and the elimination the dysfunctional organelles, respectively. Thus, the constant reshaping of mitochondria by fusion and fission referred as mitochondrial dynamics is critical to keeping under control mitochondrial quality and function (Figure 1). A failure in any of these systems predisposes to skeletal muscle dysfunction and degeneration [26]. Accordingly, alterations in mitochondrial content, morphology, and function are closely associated with muscle loss in sarcopenia and several age-related pathological conditions [26].

## 4. Mitochondrial Plasticity Declines in Aging Sarcopenia

**Figure 2:**
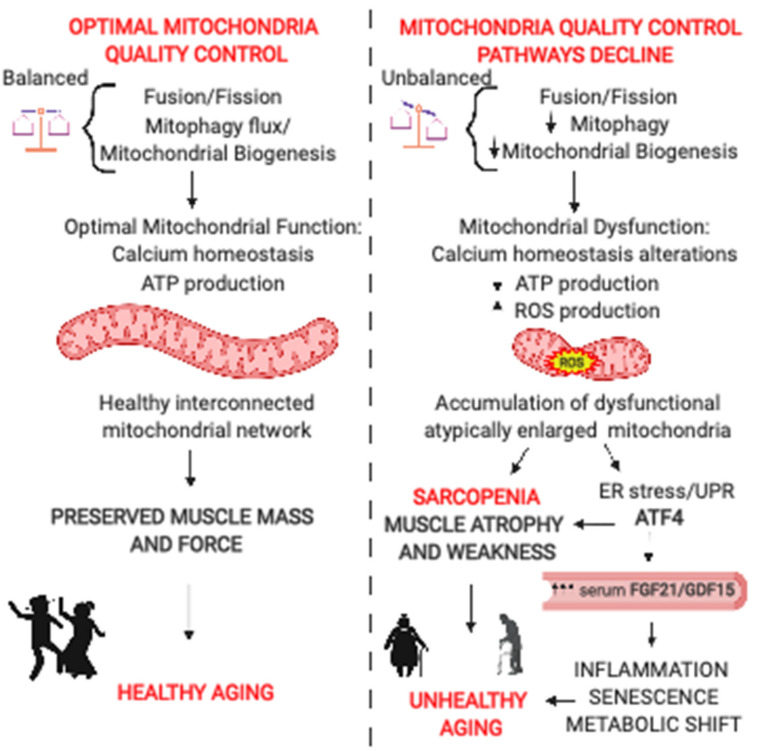
Mitochondria-derived signaling pathways controlling muscle mass and whole-body homeostasis. (**Left Panel**): Continuous rounds of mitochondrial biogenesis and fusion are balanced by opposing mitochondrial fission processes and mitophagy, leading to increased mitochondrial interconnection, optimal calcium homeostasis, and ATP production. The resulting healthy muscle mitochondrial network is critical to preserve muscle mass and force during aging. An active lifestyle is essential to preserve mitochondria and muscle function, promoting healthy aging. (**Right Panel**): The decline in mitochondria quality control pathways is a trigger of aging sarcopenia. Unbalanced fusion and fission, together with reducing mitophagy and mitochondrial biogenesis, lead to impaired mitochondrial turnover and the accumulation of dysfunctional mitochondria. Mitochondrial dysfunction in aging sarcopenia is characterized by alterations in calcium homeostasis, increased ROS production, and reduced ATP production. ROS production causes endoplasmic reticulum (ER) stress and activation of unfolded protein response (UPR). UPR induces the ATF4-dependent upregulation of fibroblast growth factor 21 (FGF21) and growth and differentiation factor 15 (GDF15) secreted by the muscle contributing to muscle loss, systemic inflammation, metabolic shift, and senescence. The combination of the resulting muscle loss and weakness and the systemic alterations promote unhealthy aging.

Loss of mitochondrial integrity results in alterations in ATP production, proteostasis, calcium handling, oxidative stress, and inflammation, which all contribute to the etiology of aging [16]. In skeletal muscle, the mitochondrial capacity to produce energy decreases with age, particularly when maximal performance is required [27]. The decline in ATP production is accompanied by enhanced ROS production, leading to further mtDNA damage and electron transport chain dysfunction that amplifies the energetic deficit [28]. Moreover, sarcopenic muscles have reduced mitochondria–sarcoplasmic reticulum calcium exchange [29], resulting in a lower capacity for mitochondrial calcium uptake in response to tetanic contraction [30]. Accordingly, mitochondrial respiration parallels muscle strength [27]. The mitochondrial functional decline in sarcopenia is linked to alterations in the mitochondrial quality control mechanisms that cause abnormal organelles’ accumulation over a lifetime, reducing mitochondrial plasticity—the mitochondrial ability to adapt to challenging conditions (Figure 2).

### 4.1. Mitochondrial Biogenesis in Aged Skeletal Muscle

In healthy cells, changes in energy requirements correlate directly with the mitochondrial content. Mitochondrial abundance is regulated by the fine-tuning of new organelle generation by mitochondrial biogenesis and removing dysfunctional organelles by mitophagy. Mitochondrial biogenesis involves the nuclear–mitochondrial coordination of transcription, translation, and import of new proteins into pre-existing organelles [31]. The coactivators PGC-1α and PGC-1β (peroxisome proliferator-activated receptor-γ coactivator-1α and β), the master regulators of the mitochondriogenesis process, are activated by stimuli that alter the cellular energy demands such as exercise, fasting, and cold exposure [32]. Because PGC-1α and PGC-1β lack DNA binding domains, they elicit their function by modulating the activity of several transcription factors, including PPARs; nuclear respiratory factors (NRFs); myocyte enhancing factors (MEFs); estrogen-related receptor (ERR); forkhead box (FoxOs); yin-yang (YY1); and transcriptional factor A mitochondrial (TFAM), the final effector of mtDNA transcription and replication [32]. In line with the age-associated mitochondrial decline, PGC1-α and -β expression decreases during muscle aging [33]. The specific deletion of PGC1-α in skeletal muscle displays a premature aging phenotype characterized by fiber damage; elevated inflammation markers; and decreased mitochondrial function, muscle force, running capacity, and balance and motor coordination [34,35,36,37]. On the other hand, muscle-specific PGC1-α transgenic mice have a delayed aging process with several features that resemble younger muscles such as the transcriptome, markers for mitochondrial function, neuromuscular junction morphology, improved calcium handling, increased autophagy markers, and decreased proteasome markers, and a slight but significant increase in lifespan [35,38].

### 4.2. Mitochondrial Morphology in Sarcopenic Muscles

Sarcopenic muscles display an alteration in the balance between fusion and fission. Aged skeletal muscle mitochondria have been reported to be fragmented in rats [39] or atypically enlarged in aged muscles of houseflies [40], mice [41,42,43], rats [44], and muscle cells of humans [41]. Morphological abnormalities in aged muscles from mice, rats, and humans depend on reduced levels of the mitochondria-shaping machinery, including the fusion proteins mitofusins 1 and 2 (MFN1 and MFN2) and optic atrophy protein 1 (OPA1), and the fission protein dynamin-related protein 1 (DRP1) [43,45,46]. Importantly, lifelong exercise counteracts the age-dependent decline of the mitochondrial machinery [45].

This section discusses how alterations in mitochondrial fusion and fission processes affect the maintenance of skeletal muscle mass and whole-body homeostasis.

#### 4.2.1. Mitochondrial Fusion and the Control of Muscle Mass and Whole-Body Homeostasis

The full knockout mice of either Mfn1, Mfn2, or OPA1 result in embryonic lethality of mice, demonstrating the biological importance of mitochondrial fusion in early development [47,48]. In humans, loss of function mutations in MFN2 and OPA1 genes cause the neurodegenerative diseases, Charcot–Marie–Tooth type 2A (CMT2A) [49] and dominant optic atrophy (DOA) [50,51], respectively. These diseases are accompanied by skeletal muscle myopathy and atrophy [52,53]. Accordingly, reducing the mitochondrial fusion machinery in muscle has been linked to age-related sarcopenia [43,45,46] and age-related metabolic diseases such as obesity and type 2 diabetes [54,55] in both rodents and humans. The simultaneous ablation of the outer mitochondrial membrane proteins MFN1 and MFN2 in skeletal muscle in mice leads to mitochondrial dysfunction, accumulation of mtDNA damage, profound muscle atrophy, growth deficit, and premature death by 6–8 weeks of age [56]. MFN2 deletion in young muscle causes muscle atrophy due to extensive mitochondrial fragmentation, mitochondrial dysfunction, ROS production, endoplasmic reticulum (ER) stress, and autophagy inhibition [43,57]. However, during aging, MFN2 deficiency exacerbates age-dependent mitochondrial dysfunction, mitophagy flux inhibition, and the accumulation of dysfunctional mitochondria, which altogether drive age-associated metabolic alterations and sarcopenia [43]. In line with the critical role of mitochondrial fusion in skeletal mass maintenance, overexpression of the inner mitochondrial membrane (IMM) profusion factor OPA1 in mice have a protective effect against acute muscle loss induced by denervation [58] as well as from chronic muscle loss in a model of mitochondrial myopathy [59]. Conversely, inhibition of OPA1, specifically in muscle, gives a similar but more severe phenotype than skeletal muscle MFN1 and MFN2 ablation [45,60,61]. OPA1 deletion in skeletal muscle during embryogenesis cause early lethality [45]. The deletion of OPA1 in the skeletal muscle of young mice causes mitochondrial dysfunction, ROS production, and mitochondrial DNA release that triggers different transcription factors such as FoxO3, NFkB, and ATF4 that coordinate the induction of a catabolic program leading to muscle loss and weakness [45,60,61]. Moreover, the muscle defects are transmitted systemically through muscle release of fibroblast growth factor 21 (FGF21), together with inflammatory cytokines such as interleukin 6 (IL6) inducing hypoglycemia, lipolysis, liver steatosis, inflammation, a pro-senescent phenotype, and premature death [45,60]. OPA1 and not MFN1 or DRP1 levels correlate with muscle mass and force loss in elderly subjects [45]. Altogether, the maintenance of mitochondrial shape and function, through fusion events, regulates muscle mass, and the crosstalk of muscle with distant organs influencing inflammation, the senescence of distant tissues, and the whole-body metabolic homeostasis.

#### 4.2.2. Mitochondrial Fission, and the Regulation of Calcium Homeostasis, Muscle Mass, and Force

Mitochondrial fission is required to maintain a healthy mitochondrial network. Impairment of mitochondrial fission leads to the disruption of mitophagy, followed by dysfunctional organelles accumulation [22,62,63,64,65,66]. Total DRP1 knockout mice are embryonically lethal, highlighting the importance of the fission machinery for tissue development and function [67]. In humans, mutations in DRP1 lead to a severe neurological syndrome with microencephaly, hypotonia, alterations in brain development, and a metabolism that causes neonatal lethality due to multi-system damage [68,69,70]. The first evidence of a causal link between mitochondrial fission and muscle maintenance comes from the observation that acute overexpression of DRP1 in muscle is sufficient to activate mitochondrial dysfunction, mitophagy, and energy stress, activating an atrophy program via the AMPK–FoxO3 axis [71]. Consistently, the constitutive overexpression of DRP1 in skeletal muscle leads to mtDNA reduction despite no alterations in mitochondrial bioenergetics, and activation of both the UPRmt and the eIF2α–ATF4–FGF21 axis, causing a reduction in protein synthesis and a blockade of growth hormones actions that prevent muscle growth [72]. While short-term inhibition of the fission machinery protects from starvation and FoxO3-dependent muscle atrophy [71], the deletion of DRP1 in skeletal muscle during embryogenesis results in reduced postnatal growth and premature lethality, and its ablation in adulthood causes muscle loss and systemic metabolic changes [62]. DRP1 inhibition display abnormal elongated mitochondria, leading to several consequences such as impaired autophagy and mitophagy, ER stress, unfolding protein response (UPR) activation, FGF21 induction, and increased MCU-dependent mitochondrial Ca2+ uptake capacity, which further reduces Ca2+ availability for contraction together with myofiber death due to calcium overload. Importantly, chronic contractile activity does not ameliorate the myopathic phenotype and neither attenuate muscle atrophy [73]. FGF21 levels can explain the observed metabolic changes, such as basal hypoglycemia, liver growth hormone (GH) resistance, and conditional knockout mice’s reduced animal size [62]. As already discussed, both an excessive activation or the impairment of fission compromise mitochondrial function and muscle health. Thus, the proper balance between mitochondrial fusion and fission is critical for muscle homeostasis. Several reports have shown that certain diseases associated with unbalanced mitochondrial fusion or fission can be rescued by rebalancing mitochondrial dynamics [59,74,75,76,77]. Like DRP1 inhibition in young muscle [62,73], aged muscles display reduced DRP1 levels, and the accumulation of elongated dysfunctional mitochondrial that cannot be properly removed due to mitophagy flux impairment [45]. Interestingly, equilibrating the unopposed mitochondrial fusion by short-term DRP1 induction in midlife *Drosophila* restored mitochondrial fission and the mitophagy flux delayed the age-onset mitochondrial damage and prolonged health and lifespan [75]. In line with the importance of restoring mitochondrial dynamics balance for muscle homeostasis during aging, the simultaneous ablation of OPA1 and DRP1 in the muscle of mice, here referred to as DKO mice, showed a less severe phenotype by mitigating age-associated features such as muscle denervation, oxidative stress, and inflammation, rescuing the lethal phenotype of muscle-specific OPA-null mice [63]. Persistent mitochondrial dysfunction activates ER stress/UPR and FGF21 pathways both contributing to muscle atrophy [78,79]. However, the atrophy program’s initial activation and the induction of FGF21 resolve over time in DKO muscle [63]. Similarly, deleting PUM2, an inhibitor of the mitochondrial fission factor (MFF), in old muscle counteracts the age-associated MFF decline, enhancing mitochondrial fission and mitophagy, and improving mitochondrial function and lifespan [74]. In conclusion, unbalanced mitochondrial dynamics are more deleterious than the simultaneous reduction of fusion and fission processes. Specific interventions aimed at re-establishing the mitochondrial dynamics balance could be critical to improving mitochondrial homeostasis and slow aging sarcopenia.

### 4.3. The Causal Role of Mitophagy Alterations in Aging Sarcopenia

Mitophagy is a cellular housekeeping mechanism to maintain mitochondrial quality in both physiological and cellular stress conditions. While mitochondrial biogenesis ensures the incorporation of new components to the pre-existing mitochondrial reticulum, irreversibly damaged organelles are separated from the mitochondrial network by the fission machinery. The defective organelles are further sequestered into autophagic vesicles for their degradation in the lysosome via the activation of mitophagy pathways [80]. In mammals, the selectivity of mitophagy is controlled by PINK1, Parkin, prohibitin 2, FUNDC1, AMBRA1, BNIP3, and BNIP3L/NIX [26]. The fine-tuning of the autophagy and mitophagy fluxes is critical for muscle mass maintenance [26]. Both excessive [71,78,81] and reduced general autophagy and mitophagy [82,83,84,85] contribute to muscle atrophy and weakness. Although some reports have shown an elevation of the mitophagy flux in aged muscles [86,87,88], several lines of evidence indicate that a decline of mitophagy efficiency during aging sarcopenia contributes to the progressive accumulation of dysfunctional organelles [89]. Accordingly, numerous mitophagy regulators decrease with age in mice and humans sarcopenic muscles [90,91,92,93], and this decrease correlates with slower walking speed in frail elderly [90], indicating a critical role of mitophagy in muscle weakness. Moreover, autophagy impairment caused by the ablation of the crucial autophagy gene Atg7, specifically in mice skeletal muscle, leads to premature aging and reduced lifespan characterized by degeneration of neuromuscular junctions, as well as increased oxidative stress, mitochondrial dysfunction, muscle atrophy, and weakness [91]. Thus, autophagy has a critical role in the maintenance of muscle homeostasis and neuromuscular junctions during aging. In agreement, impaired autophagy and mitophagy in muscles, caused by long-term mTORC1 inhibition, results in mitochondrial dysfunction and alterations in neuromuscular junctions, which are prevented by reactivating the autophagy and mitophagy flux with the autophagy activating peptide Tat-beclin1 [84]. Supporting a role for mitophagy in lifespan regulation, PINK1 and Parkin genetic deletion leads to mitophagy impairment, mitochondrial dysfunction, muscle degeneration, and decreased lifespan [94,95,96,97]. Conversely, boosting mitophagy by overexpressing PINK1, Parkin, DRP1, or the autophagy receptor p62/SQSTM1 in *Drosophila* muscles improves the age-dependent muscle function deterioration and extends lifespan [75,98,99,100]. Thus, therapeutic strategies that stimulate mitophagy may be the key to delay age-related muscle mass decline and to improve healthspan. In this respect, the pharmacological treatment with urolithin A, a metabolite from pomegranate seeds, enhances mitophagy, prevents the accumulation of dysfunctional mitochondria with age, maintains mitochondrial biogenesis and respiratory capacity resulting in extended lifespan in worms, and improves muscle force and aerobic endurance in aged mice [101]. Interestingly, a first-in-human clinical trial showed that the administration of urolithin A to aged humans induces the upregulation of mitochondrial genes in skeletal muscle, improving the molecular signature of mitochondrial and muscle health [102]. Exercise training remains the best non-pharmacological countermeasure to ameliorate age-related mitochondria and skeletal muscle dysfunction, promoting healthy aging [103]. Mitochondrial turnover and function are both altered in sarcopenic muscles and exercise training, by inducing PGC1-α, increases mitochondrial renewal and recycling by activating both mitochondria biogenesis [104] and mitophagy [105]. In conclusion, maintaining an efficient mitophagy pathway, for example, through an active lifestyle during aging or by exploring novel pharmacological approaches, is of paramount importance to preserve mitochondria and muscle function and promote healthspan.

## 5. The Role of the Myomitokines FGF21 and GDF15 in Healthy and Unhealthy Aging: the Complexity of Having a Double Life

### 5.1. Skeletal Muscle and the Regulation of Whole-Body Metabolic Homeostasis

Skeletal muscle, the largest body tissue, is a critical regulator of whole-body metabolic homeostasis. It is a major site for glucose uptake and storage as glycogen, and is the largest protein reservoir, housing 75% of all body proteins. Under nutrient depletion or metabolic stress, skeletal muscle reserves are critical to sustaining muscle metabolism and systemic energy homeostasis. Muscle is a main energy consumer, glycogen breakdown guarantees rapid energy for muscle contraction, while muscle proteolysis supports energy production elsewhere in the body by releasing amino acids that serve as gluconeogenesis substrates. Thus, skeletal muscle, by changing its mass and metabolic demand, compensates for other organs’ needs [106]. In addition to skeletal muscle indirect effect on systemic physiology, muscles directly control the body metabolism by secreting muscle-derived factors, myokines, or myometabolites, which exert autocrine, paracrine, and endocrine effects [2]. In line with muscle’s crucial importance in controlling health and disease progression in distant tissues, maintaining muscle mass by exercise promotes healthy aging [103]. In contrast, the loss of muscle mass, force, and function in aging sarcopenia are risk factors for developing age-related chronic diseases and all-cause mortality [2,8,10,11]. As stated earlier, an optimal mitochondrial function is critical for muscle homeostasis. Consequently, muscle-specific genetic interventions that enhance or disrupt mitochondrial function might feedback on total body homeostasis, delaying or accelerating systemic aging, respectively (see Section 4, Section 5.2, and Section 5.3).

### 5.2. The Myomitokines FGF21 and GDF15: The Fine Line between Health and Disease

Recent evidence suggests a critical role for FGF21 and growth and differentiation factor 15 (GDF15) in mediating the communication between skeletal muscle mitochondria and distant organs in several conditions, including aging [45,60,61,62,63,72,107,108,109]. FGF21 and GDF15 are nuclear-encoded secreting myokines that can be released in the bloodstream by several tissues, including the liver, heart, pancreas, and white adipose tissue [110,111]. Under normal physiological conditions, skeletal muscle expression levels of FGF21 and GDF15 are low. In contrast, ER and mitochondrial stress upregulate FGF21 and GDF15 expression in skeletal muscle to activate an adaptive stress response [112]. Therefore, FGF21 and GDF15 can also be defined as muscle-derived mitochondrial stress-induced factors, also named myomitokines, that induce a mitohormetic response [112]. The mitohormesis concept is associated with a dual-dose response, and probably for this reason there is still a debate as to whether FGF21 and GDF15 are beneficial or detrimental for human health. A low dose of the stress stimuli activates cell-autonomous responses to increase stress resistance and cell non-autonomous effects that improve the systemic metabolism and promote a positive effect on health and lifespan, while a higher stress stimuli dose can be detrimental [112]. Accordingly, the myomitokines FGF21 and GDF15 have been reported to have a role in health and disease progression [111,113]. FGF21 and GDF15 are induced in physiological conditions such as exercise and are associated with beneficial glucose and lipid metabolism effects [111,113]. FGF21 or GDF15 increased skeletal muscle expression play protective roles against diet-induced obesity and insulin resistance [61,108,114,115]. Moreover, FGF21 and GDF15 overexpression in mice increase longevity [116,117]. Interestingly, a mild mitochondrial uncoupling, specifically in skeletal muscle, results in increased muscle-derived FGF21 and GDF15, eliciting beneficial effects on the systemic energy metabolism, leading to healthy aging and increased lifespan [108,109,118,119]. The beneficial systemic metabolic effects on metabolic flexibility and insulin sensitivity depend on the diurnal action of GDF15 [108], while FGF21 signaling is dispensable [120]. However, inhibition of either FGF21 or GDF15 prevented the browning of the white adipose tissue [108,120]. Conversely, FGF21 and GDF15 have been proposed as markers of mitochondrial myopathies [121,122] and aging [45,107,123], with levels associated with worsening health parameters and reduced life expectancy in older people [107]. Moreover, higher FGF21 and GDF15 circulating levels are associated with age-related diseases such as obesity, cardiovascular disease, insulin resistance, type 2 diabetes, and neurodegeneration [111,113], as well as cancer cachexia [124,125]. The inhibition of GDF15 activity, by targeting its receptor GDNF family receptor alpha-like (GFRAL) in brainstem neurons of tumor-bearing mice, identified a novel strategy for cancer cachexia treatment. This approach preserves weight loss independently of food intake, white adipose tissue, and muscle force and reverses tumor-induced muscle wasting by reducing several atrophy-related genes such as Atrogin1, Gadd45α, and Bnip3 [125]. The cachexia-induced muscle loss protection suggests a role for GDF15 in muscle mass maintenance. Moreover, GDF15 muscle and serum levels inversely correlate with muscle cross-sectional area in chronic obstructive pulmonary disease (COPD) patients [126] and with muscle force in lower limb mobility impairments [127]. Moreover, the overexpression of GDF15 in muscle is sufficient to induce muscle atrophy [126], likely by FoxO1 and SMAD3 activation [128]. However, it is unclear whether GDF15 effects on muscle catabolism occur through the GFRAL receptor because there is no consensus regarding its skeletal muscle expression in physiological conditions [128,129], and muscle GFRAL levels under catabolic conditions have not been investigated yet. In contrast, FGF21 and its co-receptor β-Klotho and its receptors FGFR1b, FGFR1c, and FGFR4 are highly expressed in skeletal muscle under catabolic conditions, suggesting FGF21 cell-autonomous effects [45,78,130]. Accordingly, we have recently identified a novel role for FGF21 in the control of muscle mass. FGF21 is sufficient and required to activate muscle atrophy by activating Bnip3-dependent mitophagy pathways [78]. Thus, FGF21 and GDF15 are commonly induced when mitochondrial function is impaired in skeletal muscle to amend ongoing stress. However, FGF21 and GDF15 contribution to beneficial or pathological outcomes depends on the myomitokines’ capacity to overcome the stress condition and restore homeostasis.

### 5.3. The Link between Skeletal Muscle Mitochondrial Shape, FGF21, GDF15, and Aging

Alterations in skeletal muscle mitochondrial dynamics not only induce muscle atrophy and weakness, but it can also affect the whole-body metabolic homeostasis and aging. The resulting mitochondrial dysfunction can activate a retrograde response from mitochondria to the nucleus, resulting in the secretion of the myomitokines FGF21 and GDF15 that mediate an adaptive response to mitochondrial stress. As previously discussed, different animal models have shown that either unbalanced mitochondrial fusion or fission in skeletal muscle cause mitochondrial dysfunction, ER stress, and UPS activation, resulting in muscle and serum FGF21 increased levels (see Section 4.2). However, senescence and animal survival are different. For example, OPA1 inhibition in skeletal muscle results in accelerated aging and premature death [45], muscle-specific DRP1 null mice display a normal lifespan [62], while the simultaneous ablation of OPA1 and DRP1 in muscle mitigates age-associated features and rescues the lethal phenotype of OPA1 [63]. These differences can be in part explained by FGF21 dose-dependent effects, where a mild or transient induction results in adaptive responses to stress, while a dramatic or chronic increase overcomes the beneficial effects becoming detrimental [107,112]. Accordingly, FGF21 serum levels are lower in DRP1 knockout mice [62] than OPA1-null mice [45,60], and GDF15 and FGF21 serum levels are only transiently elevated in DKO mice [63]. Supporting a threshold effect of the myomitokines, a mild inhibition of OPA1 in muscle does not alter mitochondrial complex and supercomplex formation, thus inducing lower muscle-derived FGF21 serum levels than in the model where OPA1 is completely deleted in adulthood [45], resulting in FGF21-mediated beneficial metabolic changes in terms of resistance to diet-induced obesity [61]. Because FGF21 and GDF15 high circulating levels are associated with both beneficial effects and pathologic conditions, it is unlikely that these myomitokines are detrimental per se. Therefore, they cannot be considered biomarkers of disease alone. Accordingly, the different effects of FGF21 and GDF15 on aging and survival can also be explained by considering that the inflammatory response induction might synergize with FGF21 and GDF15 in senescence induction. OPA1 inhibition triggers IL6 and IL1 upregulation via ROS [45,60], and anti-inflammatory treatment is sufficient to reduce FGF21 muscle and circulating levels [60] while the simultaneous inhibition of OPA1 and DRP1 prevents the inflammatory response [63], and the ablation of DRP1 does not alter the expression of inflammatory cytokines [62]. Taken together, in response to alterations in mitochondrial morphology and function in skeletal muscle, the combination between acute and transient versus chronic and persistent FGF21 and GDF15 myomitokines together with synergizing or antagonizing factors determine a healthy or unhealthy outcome on aging and survival.

## 6. Conclusions

Skeletal muscle is a major determinant of life quality, contributing to a healthy or unhealthy aging outcome. The maintenance of a functional mitochondrial network is critical for preserving skeletal muscle homeostasis throughout the lifespan. Accordingly, a failure in the pathways controlling mitochondrial quality is a major mechanism triggering aging sarcopenia. Alterations in mitochondrial fusion and fission events and reduced mitochondrial turnover cause abnormal organelles accumulation over a lifetime, resulting in reduced mitochondrial function and plasticity that impinges on the activation of catabolic pathways leading to muscle loss and weakness. Notably, muscle mitochondrial dysfunction is not confined only to the muscle fiber but can be transmitted systemically through the muscle-release of the myomitokines FGF21 and GDF15. Recent research has uncovered the complexity of FGF21 and GDF15 biological roles in both beneficial and pathological processes that result in delaying or accelerating systemic aging. FGF21 and GDF15 action can be affected by synergizing or antagonizing factors, FGF21 and GDF15 circulating levels that can elicit negative effects when reaching a certain threshold, and acute and transient versus chronic and persistent FGF21 and GDF15 secretion. Thus, better knowledge on the factors enhancing or counteracting FGF21 and GDF15 effects and how these factors combine in a specific context could help to understand when FGF21 and GDF15 can be used to enhance their beneficial effects to promote healthy aging and when they should be modulated to counteract unhealthy aging.
